# A Clinical Case

**Published:** 1882-11

**Authors:** J. W. Thompson

**Affiliations:** Springfield, Mass.


### A CLINICAL CASE.

J. W. THOMSON, M. D., SPRINGFIELD, MASS.

Case Book No. 5. Page 34. Miss E. A., æt. eleven years and nine months. Height, 5 ft. 3 in. Weight, 80 lbs. Was brought to my office March 23d, 1882. Intense photophobia; left eye worst. Dreads artificial light more than daylight. Is myopic. Left eye smarts. Sclerotica of both eyes congested and inflamed. Small chemotic patches on both eyes. Lids granulated, and of a deep mahogany color. Sometimes—not often—has headache through forehead, over eyes. Tongue unusually large and thick, having a slightly white coat. On exercise, sharp pain in hepatic region. Breath is very offensive. Sleep, breathes very hard during, and snores. Last joints of both little fingers and toes wanting; no nails, *i. e.*, on little fingers and toes. Drinks a great deal of cold water; takes it to her room, and drinks before retiring. Pulse fine, tense and wiry, 112. ℞. Bell. 30th, twice daily for a week.

*Note.*—I call the Belladonna 30th; that is what it was about ten years ago. I have refilled it with deodorized alcohol, probably from forty to fifty times, to medicate pellets, sò that it is, in all likelihood, nearly the 100th potency.

The young lady was brought to my office on the first of the following month. She had improved wonderfully. The spots of chemosis were no longer visible. Instead of the intense redness of the lids, only a slight tinge. The smarting of the left eye had departed for parts unknown. Did not snore nearly so much. Her mother remarked that she slept quieter and easier on the first night after taking the medicine. Has not had the sharp pain in liver. Pulse softer, 102. The myopic condition unchanged, but can read without hurting her eyes.

